# Food for Young and Old

**Published:** 1888-01

**Authors:** 


### FOOD FOR YOUNG AND OLD.

After a child's first teeth come, try him with bread and milk, then cautiously, oatmeal, rice, wheat and similar grain foods, one at a time as you find them able to digest them. There should be no great change of food attempted in July or August. Whatever agrees with a year old child in June should be continued the following two or three months. A lady who believed this, carried her plump, rosy, but teething baby through the second summer in a city boarding house on three meals a day, of bread and milk alone, without an idle day. But her hard heartedness afforded a constant topic to her fellow boarders.

It is possible, in winter, to feed a child who is more than a year old, some fruit, but this should be suspended for the hot season. In case of an elder child, a little of one kind of fruit, when eaten in the morning rarely sickens. But fruit should never be given after three o'clock in the afternoon. Many a sick night follows a too hearty or too varied supper, and the plainer the last meal of the day, the better.

This diet may seem needlessly plain, but we must remember that little babies have not our cultivated appetites, and do not crave variety as much as we do. No child needs the stimulus of meats until he is three or four years old, unless in the form of weak broths. Vegetables, except potatoes, and not too much of that and never fried, are wholly unsuited to a young child's stomach. So are pastry, shortened cakes and rich cookies. Gingerbread is good but somewhat treacherous.

It is true some children do live through all kinds of meals, at all hours, but in regular hours and plain diet is the only safety for many. That the few live, only proves how long suffering is nature. The connection between life and nutrition is so close and inseparable, that the continuance of the former depends upon the supply of the latter. The variation and form of life, whether animal or vegetable, depends upon the quantity and quality of nutrition with which it is supplied.

We are readily able to judge of the quantity and quality of nourishment supplied to a vegetable that we are caring for, and will at once decide whether the supply is normal, insufficient or in excess of the nutrition necessary. Every living organism demands its own peculiar supply designed for it by the laws of nature. When the natural supply is normal in all respects, the life is healthy. The same law holds good in the higher order of life that we observe in the lower. Insufficient alimentation in the vegetable kingdom is followed by consequences which we are not liable to misunderstand. There is less of health and vigor, a gradual shrinking, and, if the process is permitted to go on, death occurs. A great excess of even the natural nutrition tends to the same result. A marked departure in either direction from the normal standard of supply will, with very few exceptions, be followed by a marked departure from the normal standard of life.

It is needless to mention that brutes suffer from improper feeding and particularly, over-feeding. Excessive alimentation in the human being is followed by the same baneful consequences. Is it not a fact that the generality of people eat too much? Very few eat too little. It is a commonplace aphorism: "He eats so much that it makes him poor to carry it." He who eats more than is demanded by nature, imposes a heavy strain on the organs involved in the process of digestion and elimination. The stomach, for instance, is capable of doing a certain amount of work, hence, if over-taxed unduly, the result, sooner or later, will be imperfect digestion, impure blood, disordered functions and gradual loss of vigor and strength. As certain as we overstep the bounds of nature and overtax our stomach for our palate's sake, we begin to sow the seeds of disease. Certainly, the stomach is a remarkable organ, and able to resist the assaults of imprudence with wonderful courage, but its power of endurance will not withstand every imposition heaped upon it, and sooner or later, it will succumb to the force of the unrelenting overwork.

There is far more danger from over-indulgence than under-eating.

